# Overlooked electrolyte destabilization by manganese (II) in lithium-ion batteries

**DOI:** 10.1038/s41467-019-11439-8

**Published:** 2019-07-31

**Authors:** Cun Wang, Lidan Xing, Jenel Vatamanu, Zhi Chen, Guangyuan Lan, Weishan Li, Kang Xu

**Affiliations:** 1 National and Local Joint Engineering Research Center of MPTES in High Energy and Safety LIBs, Engineering Research Center of MTEES (Ministry of Education), and Key Lab. of ETESPG(GHEI), South China Normal University, 510006 Guangzhou, China; 2 Electrochemistry Branch, Sensor and Electron Devices Directorate, Power and Energy Division, U.S. Army Research Laboratory, Adelphi, MD 20783 USA

**Keywords:** Electrochemistry, Batteries

## Abstract

Transition-metal dissolution from cathode materials, manganese in particular, has been held responsible for severe capacity fading in lithium-ion batteries, with the deposition of the transition-metal cations on anode surface, in elemental form or as chelated-complexes, as the main contributor for such degradations. In this work we demonstrate with diverse experiments and calculations that, besides interfacial manganese species on anode, manganese(II) in bulk electrolyte also significantly destabilizes electrolyte components with its unique solvation-sheath structure, where the decompositions of carbonate molecules and hexafluorophosphate anion are catalyzed via their interactions with manganese(II). The manganese(II)-species eventually deposited on anode surface resists reduction to its elemental form because of its lower electrophilicity than carbonate molecule or anion, whose destabilization leads to sustained consumption. The reveal understanding of the once-overlooked role of manganese-dissolution in electrolytes provides fresh insight into the failure mechanism of manganese-based cathode chemistries, which serves as better guideline to electrolyte design for future batteries.

## Abstract (web summary)

Mn dissolution is dominantly responsible for capacity fading of most Mn-rich cathodes. Here the authors reveal that soluble Mn^2+^ species significantly destabilizes solvent and anion via its unique solvation sheath structure, providing insight into the failure mechanism of related cathode chemistries.

## Introduction

Manganese-rich (Mn-rich) cathode chemistries attract persistent attention due to pressing needs to reduce the reliance on cobalt in lithium-ion batteries (LIBs)^1,2^. Recently, a disordered rocksalt material with reversible Mn^2+^/Mn^4+^ redox-pairs was proposed, promising extremely high capacity of >300 mAh g^−1^ and energy density of ~1000 Wh kg^−1^. Such new materials created opportunities for the next generation LIBs, but will inevitably encounter the decade-long challenge of Mn^2+^-dissolution in electrolytes, which has prevented the application of almost all Mn-rich materials^3^. Dissolution of transition-metal (TM) ions is closely associated with battery performance-degradation^4–11^, the accepted mechanism behind which has been overwhelmingly focused on interfacial TM-species on anode surface, resulting from dissolved TM-ions migrating from cathode and eventually depositing, in either elemental form or chelated TM-complexes, as part of the solid-electrolyte-interphase (SEI) on anode^12–17^. However, the role of soluble Mn^2+^-species in bulk electrolytes has been completely overlooked thus far. Given the continuous Mn^2+^-dissolution and its slow migration to the anode surface, Mn^2+^-species should accumulate to sufficiently high concentrations in bulk electrolyte, and it is of high interest to understand how the bivalent Mn^2+^ as a rival cation of Li^+^ competes for the solvation from both carbonate and anion. As established earlier by Xu and coworkers that Li^+^-solvation directly dictates interphasial chemistries in LIBs^18,19^, the competitive solvation between Li^+^ and Mn^2+^ should never be under-estimated.

Here in this work, using computation, spectroscopic, and electrochemical characterizations, we identify the dissolved Mn^2+^ as catalytic center that induce continuous electrolyte consumption at the expense of the cell performances. In comparison with Li^+^, the bivalent Mn^2+^ forms larger solvation sheath containing both PF_6_^−^ and carbonate molecules, wherein Mn^2+^ readily activates these solvation members either for side reactions in the bulk electrolyte or for electrochemical reductions at anode surface. This destabilization becomes overwhelming at elevated temperatures, leading to significant change in both bulk electrolyte composition and interphasial chemistry. Such precise understanding of how soluble Mn-species affect battery performance constitutes critical knowledge to enable these Mn-rich cathode chemistries.

## Results

### Mn^2+^-solvation structure in carbonate-based electrolyte

The ability of Mn^2+^ to compete for the solvation from carbonate solvents and anions and the resultant solvation sheath structure were calculated by density function theory (DFT) (Supplementary Fig. 1), in comparison with the interacting structures and energy of Li^+^. The calculated interaction energies of Mn^2+^ with ethylene carbonate (EC), dimethyl carbonate (DMC), ethyl methyl carbonate (EMC), diethyl carbonate (DEC), PF_6_^−^, and bis(trifluoromethane sulfonyl)imide (TFSI^−^) are −51.11, −41.49, −43.17, −43.97, −45.39, and −72.37 (with O in TFSI^−^) kJ mol^−1^, respectively. Here TFSI^−^, which does not contain chemically labile fluorine bonds and is intrinsically non-acidic, was used as a reference with the acidic anion PF_6_^−^ typically used in state-of-the-art electrolytes.

**Fig. 1:**
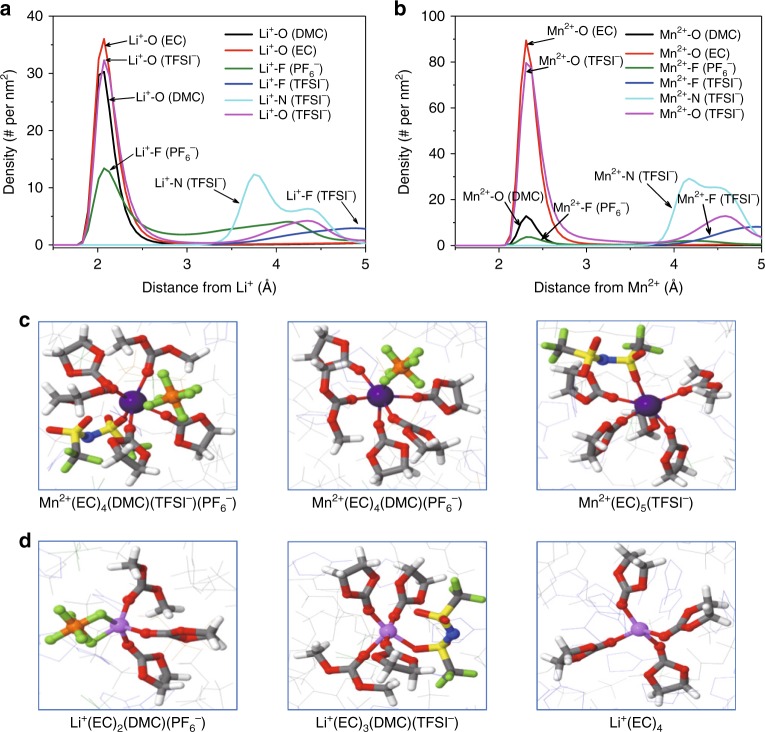
Electrolyte structures obtained from molecular dynamic simulation. Radial distribution function for Li^+^ (**a**) and Mn^2+^ (**b**) in electrolytes; The typical first solvation sheath structures of Mn^2+^ (**c**) and Li^+^ (**d**); Part of these structures that exist in liquid solutions can be detected by ESI-MS in gaseous state after partial desolvation of the cations

The interaction energies of Mn^2+^ are found to be constantly higher (more negative) than those of Li^+^, which unambiguously indicate that the bivalent cation Mn^2+^ enjoys the privilege of being solvated by carbonate molecules or anions in competition with Li^+^ (Supplementary Fig. 1). Equally important is the preference of EC and TFSI^−^ by Mn^2+^ over the other investigated carbonate solvents (DMC, EMC, and DEC) and PF6^−^ anion. A Mn^2+^-solvation sheath at equilibrium, therefore, should be mainly populated by EC and TFSI^−^. Indeed, differing from Li^+^ (Fig. 1a), the radial distribution function obtained from molecular dynamic (MD) simulation reveals that the first solvation shell of Mn^2+^ is dominated by carbonyl O in EC, O in TFSI^−^, and to a much lesser extent by F in PF_6_^−^ and carbonyl O in DMC (Fig. 1b). Due to its higher charge density and roughly the same ionic radius (+2 and 80 pm for Mn^2+^ vs. +1 and 90 pm for Li^+^), Mn^2+^ requires higher solvation number than Li^+^ in order to remain soluble, hence its first solvation sheath is larger than Li^+^ as identified from MD simulations (Fig. 1c, d). It is worth mentioning that DFT calculations and MD simulations respectively identified N (Supplementary Fig. 1) or O (Fig. 1b, c) in TFSI^−^ as the main solvating site for Mn^2+^. MD simulation should be closer to reality, because with lower steric hindrance, O in TFSI^−^ is expected to be more accessible to Mn^2+^, which in turn generates a larger solvation sheath.

As TFSI^−^ is not a typical salt anion used in most commercial electrolytes and only introduced as a non-acidic anion reference to evaluate the decomposition effect of PF_6_^−^, how PF_6_^−^ and EC interact with Mn^2+^ is of primary interest. Surprisingly, the length of F^−^P bond in PF_6_^−^ increases from 1.62 to 1.77 Å (Supplementary Fig. 1) when interacting with Mn^2+^, as compared with 1.68 Å when interacting with Li^+^. Similar trend can also be obtained from MD simulations (Supplementary Fig. 2a), suggesting that Mn^2+^-dissolution into PF_6_^−^-containing electrolyte would result in Mn^2+^-solvation sheaths where PF_6_^−^ anion becomes rather activated as indicated by the longer P-F bond. The torn-apart PF_6_^−^ subsequently generates PF_5_, an especially strong Lewis acid, which has been known to trigger a series of carbonate molecule decompositions^20^. In the reaction profile of EC (Supplementary Fig. 2b), the presence of PF_5_ greatly reduces the decomposition energy barrier by 39 kJ mol^−1^. Such catalytic effect would be exponentially accelerated at elevated temperatures.

### Influence of dissolved Mn^2+^ on electrolyte thermostability

**Fig. 2:**
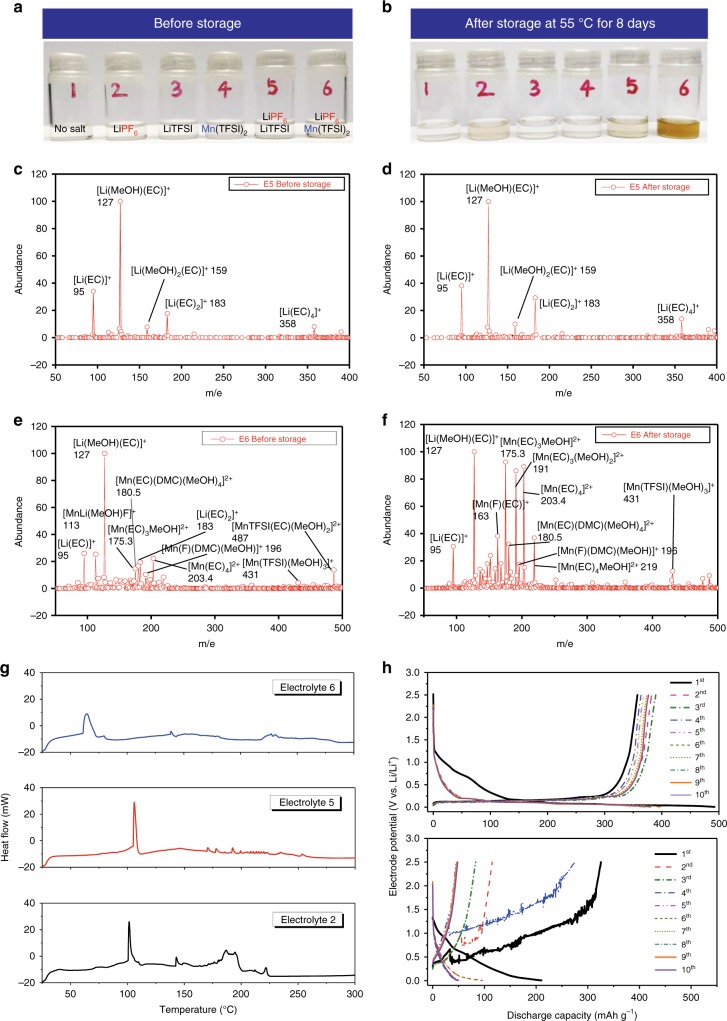
Presence of Mn^2+^ ions triggers thermal decomposition of electrolyte. Electrolyte discoloration before (**a**) and after (**b**) storage at 55 °C for 8 days; ESI-MS characterizations of Electrolytes 5 and 6 before (**c**, **e**) and after (**d**, **f**) storage, where methanol was used as diluent solvent; DSC curves of fresh Electrolytes 2, 5, and 6 (**g**); charge/discharge voltage profiles of graphite/Li half-cells containing Electrolyte 6 before (upper, **h**) and after thermal storage (lower, **h**) at a current density of 74.5 mAh g^−1^ (0.2 C) between 0.005 to 2.5 V vs. Li/Li^+^

Experiments firmly support the above theoretical calculation regarding the unique solvation sheath structure of Mn^2+^ and how this structure destabilizes electrolyte components. Electrolytes containing both Li^+^ and Mn^2+^ cations with PF_6_^−^ and TFSI^−^ anions were prepared and labeled Electrolytes 1 through 6 (Fig. 2a, b, and Supplementary Table 1), which were then subject to storage at 55 °C for 8 days, so that potential side reactions are accelerated in a visible manner. The color of LiPF_6_-free electrolytes (Fig. 2a, b, Electrolytes 1, 3, and 4) remain unchanged after such storage, indicating that neither Li^+^, Mn^2+^ nor TFSI^−^ will induce drastic decomposition. Under the same condition, the two electrolytes containing LiPF_6_ (Electrolytes 2 and 5) discolor slightly, identifying PF_6_^−^ as the source that induces the side reactions of carbonate-based electrolyte to a moderate extent^21,22^, perhaps via the trace presence of PF_5_. In sharp contrast, significant color-change occurred when Mn^2+^ and PF_6_^−^ coexist (Electrolyte 6), which directly confirms the destabilization of the electrolyte via the cooperation of both Mn^2+^ and PF_6_^−^. The measured average H_2_O content for Electrolytes 1, 2, 3, 4, 5, and 6 before storage is 21, 24, 65, 70, 68, and 72 ppm, respectively. The TFSI-containing electrolytes (Electrolytes 4, 5, and 6) show higher moisture than the others due to the commercial TFSI-salts (both Li^+^ and Mn^2+^) which were synthesized via aqueous routes, and whose moisture level has always been an issue. However, it should be noted that the discoloration of Electrolyte 6 (containing combination LiPF_6_/Mn(TFSI)_2_) is significantly more obvious than both Electrolytes 4 and 5 (containing either Mn(TFSI)_2_ alone or combination of LiPF_6_/LiTFSI; Fig. 2b), despite their H_2_O contents being essentially the same within experimental error. This clear distinction decouples the discoloration of Electrolyte 6 from moisture content, and strongly suggests that the electrolyte degradation of number 6 should be ascribed to the catalytic effect of Mn^2+^ and its interaction with PF_6_^−^ anion. To further validate this conclusion, 1000 ppm HF was injected into electrolytes and then stored at 55 °C for 8 days (Supplementary Fig. 3). The HF injection caused obvious discoloration as compared with the electrolyte without HF injection (Fig. 2b), indicating that HF indeed also causes electrolyte decomposition. However, the discoloration trend after storage (Supplementary Fig. 3) remains the same with that shown in Fig. 2b, with Electrolyte 6 being the most severe. This further confirms that the decomposition of Electrolyte 6 is firmly correlated with the co-existence of Mn^2+^ and PF_6_^−^.

Electrospray ionization mass spectrometry (ESI-MS) was used to map the solvation sheath structures of both Li^+^ and Mn^2+^. While established earlier as a precise and quantitative tool that reveals the distribution of solvent molecules around Li^+^, it should be cautioned that the cation complexes as detected by ESI-MS in gaseous state actually already experienced partial desolvation during the flight under electric field, therefore, these charged species do not directly reflect the exact solvation sheaths in electrolyte solution. However, they do represent the most stable solvation species that should occur in the electrolyte solution^18^. It is apparent that in electrolyte containing only Li salts (Electrolyte 5), Li^+^ is preferentially solvated by EC molecules (Fig. 2c), as evidenced by the pronounced peaks at mass-charge ratios (m/e) of 95, 127, 183, and 358 that correspond to [Li(EC)]^+^, [Li(MeOH)(EC)]^+^, [Li(EC)_2_]^+^, and [Li(EC)_4_]^+^, respectively, despite that EC and DMC coexist at almost equimolar ratio (EC/DMC = 1.02). Methanol (MeOH) was used as diluent solvent in the ESI-MS experiments, hence solvation sheaths containing MeOH also persistently appears, showing that MeOH is a strong competitor against EC in solvating Li^+^. After this electrolyte underwent thermal storage, its ESI-MS still maintained a rather clean spectrum with almost identical patterns of these same solvation species (Fig. 2c, d), confirming the essential stability of electrolyte when PF_6_^−^ was the only anion therein.

On the other hand, upon addition of Mn^2+^, a host of new species immediately appeared even before high temperature storage (Fig. 2e), as evidenced by the rather noisy background. Although it is difficult to identify all these new species, which are apparently the directly result from the electrolyte decomposition catalyzed by Mn^2+^ at room temperature, it is beyond doubt that extensive and chaotic reactions already occurred. Closer examination reveals that, aside from a few Li^+^ species (m/e 95, 127, 183), Mn^2+^-solvation species now exist, such as [Mn(EC)_3_(MeOH)]^2+^ (m/e 175.3), [Mn(EC)(DMC)(MeOH)_4_]^2+^ (m/e 180.5), and [Mn(EC)_4_]^2+^ (m/e 203.4). TFSI^−^ can also be observed in Mn^2+^-solvation as [MnTFSI (MeOH)_3_]^+^ (m/e 431) and [MnTFSI(EC)(MeOH)_2_]^+^ (m/e 487), whose presence, despite the low relative abundances, offers a firm support for the simulation result that TFSI^−^ anion is preferred by Mn^2+^. On the other hand, no Mn^2+^-solvation sheaths that contain PF_6_^−^ anion can be detected in Fig. 2e, consistent with the calculation results PF_6_^−^ in Mn^2+^-solvation sheath will be destabilized, leading to various fragments including F^−^, PF_5_ or other degraded compounds. The presence of [Mn(F)(DMC)(MeOH)]^+^ at m/e 196 serves as a further evidence that PF_6_^−^ reacts with Mn^2+^ even before storage.

Storage at 55 °C significantly accelerates these decompositions, whose ESI-MS (Fig. 2f) becomes dominated by Mn^2+^-solvation species, in sharp contrast with the spectra before the storage (Fig. 2e) where Li^+^-solvation species still prevailed. More importantly, it now display a much more complicated background pattern that reflects extensive decomposition, among which the prominent species include the Mn-containing species consisting of the fragments of electrolyte components such as [MnF(DMC)]^+^ (m/e 164), [MnF(MeOH)_2_] ^+^ (m/e 137.9) and [MnF(DMC)(MeOH)_2_]^+^ (m/e 196), etc. Apparently, at this moment the bulk composition of the electrolyte is no longer what represented by the face formula of [1.0 M LiPF_6 + _0.3 M Mn(TFSI)_2_ in EC/DMC]. Instead, it contains a high Mn^2+^-concentration accompanied by a series of decomposition products from PF_6_^−^ anion, including F^−^ and other fragments resulting from the carbonate molecules. Aside from liquid phase, analysis was also performed on the gaseous products resulting from the electrolyte decomposition catalyzed by Mn^2+^-species. Thus, Electrolytes 5 and 6 were stored in pouch bags and left at 55 °C for 8 days, after which ~0.8 mL of gas samples were extracted from each bag. The co-existence of Mn^2+^ and PF_6_^−^ (Electrolyte 6) apparently induced significant decomposition reactions (Supplementary Fig. 4), as evidenced not only by the numerous peaks with varying retention time, but also by the significant quantity as reflected by peak 7. While it is impossible to accurately identify each species due to the chaotic decomposition reactions, which are further complicated by the fragmentation process in MS, we can recognize diversified F-containing species that have origin from the carbonate esters. Most importantly, the detailed analysis on the low-retention time peaks (*t* < 7 min) identified a dominant species at m/e = 69, which should be attributed to [PF_2_]^+^, most likely originated from PF_6_^−^ or PF_5_ by Mn^2+^ then fragmented during the bombardment in MS stage.

Differential scanning calorimetry (DSC) performed on electrolytes displays a major exothermic reaction at 64 °C for Electrolyte 6, which is ca. 42 °C lower than the other two electrolytes without Mn^2+^ (Electrolytes 2 and 5; Fig. 2g). This process should correspond to the spontaneous decomposition reactions initialized by Mn^2+^ and participated by PF_6_^−^ anion and carbonate solvents. Although most LIBs operate at ambient temperatures, it should be mentioned here that the core temperature of the spirally wound cells could easily surpass 40 °C, while high rate drain drives such temperature even higher to the neighborhood of these above exothermic onset^23^. In other words, the above electrolyte decompositions induced by Mn^2+^ could exist universally. Unfortunately, this potentially critical failure mechanism has been overlooked thus far when evaluating how Mn dissolution induces performance degradations of LIBs.

Acidity measurement provides a supplemental way to quantify how electrolytes decompose, as it was well known that HF-content in an electrolyte is proportionate to PF_5_, which is more sensitive to hydrolysis by trace moisture than does PF_6_^−^ anion^24,25^. This is exactly what Supplementary Fig. 5 indicates, i.e., high acidity arises from Mn^2+^-containing electrolyte after thermal storage. As the initial H_2_O contents of Electrolytes 5 and 6 are essentially the same within experimental error, the higher acidity of Electrolyte 6 after storage should be mainly ascribed to the decomposition of PF_6_^−^ induced by the presence of Mn^2+^. Meanwhile, the Mn^2+^-presence significantly impacted the cycling stability of the LIB, as shown in Fig. 2h (upper) and Supplementary Fig. 6, where graphite/Li half-cells were assembled using Electrolyte 6 both before and after thermal storage. The reversible lithiation/delithiation of graphite could still be supported by Electrolyte 6 before high temperature storage, despite the significant decomposition of bulk electrolyte catalyzed by Mn^2+^-species as represented by the event at ~1.0 V vs. Li in the 1st lithiation process. This irreversible event could be more visually outstanding when overlaid with Electrolyte 5 that contains no Mn^2+^ (Supplementary Fig. 7), and should correspond to the electrochemical reduction of EC molecules activated in the Mn^2+^-solvation sheath. Thermal storage at elevated temperature further catalyzed these decomposition reactions to a point where the bulk electrolyte composition is no longer what the face formula represents, and the graphite cannot even be lithiated/de-lithiated for a single cycle now (Fig. 2h, lower). In fact, the delithiation process of graphite in Electrolyte 6 after storage displays a rather abnormally high “capacity” at a potential that positively deviates from the normal delithiation profile of graphite anode. This parasitic process was caused by the electrochemical reactivity of the side products generated by Mn^2+^-catalysis during the storage. Those impurities, most of which were captured by the ESI-MS for Electrolyte 6 after storage (Fig. 2f), react with both graphite anode and Li metal counter electrode. Especially during Li-deposition (or delithiation of graphite), the nascent Li-deposition presents a far more reactive surface on Li metal, inducing severe reductions. Such “asymmetry” creates a pseudo-excessive delithiation capacity as observed in Fig. 2h. Post-mortem analysis on graphite conducted by scanning electron microscope (SEM), elemental mapping and transmission electron microscope (TEM) confirm the massive electrolyte decomposition in Electrolyte 6, which leads to significant population of O-containing species that almost completely camouflaged the presence of C and Mn on the graphite surface (Supplementary Fig. 8).

### Effect of deposited Mn-species on electrolyte stability

**Fig. 3:**
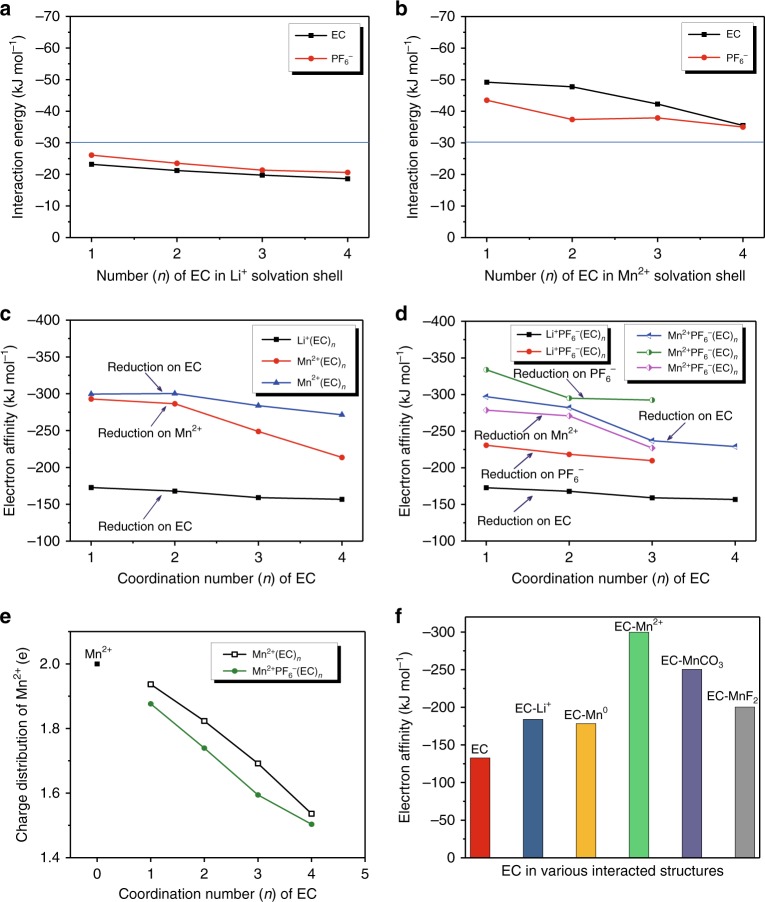
Interaction energy and reduction activity of Mn^2+^ species. Interaction energy of EC solvent and PF_6_^−^ with Li^+^ (**a**) and Mn^2+^ (**b**); Electron affinity energy of Li^+^ and Mn^2+^ solvation shells with (**c**) and without (**d**) PF_6_^−^; Charge distribution of Mn^2+^ in solvation shells (**e**); Electron affinity energy of EC in various interacted structures (**f**)

Aside from its effect on the stability of bulk electrolyte components, Mn^2+^ also affects the interfacial reactions when its solvation sheath approaches the anode surface. Considering that the interaction energies of Mn^2+^ with both EC and PF_6_^−^ are much higher (more negative) than Li^+^, it is logical to anticipate that Mn^2+^-solvation sheath alters how the Li^+^-solvation sheath structure dictates interphasial chemistry as established previously^26^. Specifically, in an electrolyte where PF_6_^−^ is the only anion, the binding between the 4^th^ EC solvent and [Mn^2+^(EC)_3_PF_6_^−^] or between PF_6_^−^ and [Mn^2+^(EC)_4_] is much stronger than those between EC and [Li^+^PF_6_^−^] ion-pair or PF_6_^−^ and [Li^+^(EC)] (Fig. 3a, b). Hence a Mn^2+^-solvation sheath would be much more difficult to desolvate than a Li^+^-solvation sheath. As consequence, the “kidnaped” anion may very likely be brought to the anode surface by Mn^2+^ and experience the low potential there. This would significantly raise the opportunity of anion-reduction and introduction of anion-originated species as part of the new interphase. The one-electron reduction activity of Li^+^ and Mn^2+^ solvation sheaths with and without PF_6_^−^ anion were thus compared in Fig. 3c, d. The optimized structure of Mn^2+^ solvation sheaths before and after one electron reduction were shown in Supplementary Fig. 9, with the structures of Li^+^ solvation sheath already reported in our previous work^26^. The electron affinity energy of Mn^2+^-solvation sheath is found to be significantly higher (negative) than that of Li^+^-solvation sheath, indicating the former (Mn^2+^) should be the winner in the competition for electron from anode surface over the latter (Li^+^), in good agreement with the conclusion drawn earlier that Mn^2+^-presence in electrolyte accelerates the electrolyte reduction^27^.

Interestingly, for a Mn^2+^-solvation sheath without PF_6_^−^, the gained electron prefers to associate with EC molecule instead of occupying the 3d orbital of Mn^2+^ (Fig. 3c), implying that EC will be preferentially reduced over Mn^2+^. If the Mn^2+^ solvation sheath contains PF_6_^−^, however, the gained electron prefers to associate with PF_6_^−^ (Fig. 3d), leading to a series of fluorides [PF_5_ and (MnF)^+^], which are even more reactive toward carbonate molecules than the salt anion itself. Considering that both types of Mn^2+^-solvation sheaths coexist as evidenced by ESI-MS data (Fig. 2e, f), the above processes should proceed simultaneously. In either case, the reductive pathway of Mn^2+^-species on anode surface does not generate Mn^0^, but instead catalyzes the reduction of the solvating members within the Mn^2+^-solvation sheath, be it carbonate molecules or anion. This conclusion agrees with the recent observations by Lu et al. and Jarry et al. respectively that TM cations retain their oxidation state on anode surface^28–30^, and directly rectifies the early belief that they will be reduced into elemental form on anode surface because of their higher redox potential (e.g., Mn/Mn^2+^ at 1.87 V vs. Li/Li^+^)^4,27^. We believe that this early misconception might have arisen because the critical role of solvation sheath was overlooked. As in the scenarios displayed by Xu and coworkers for Li^+^, these solvation sheaths serve as molecular-sized reaction vessels, in which the central cation activates their solvation member and directs how the reaction should proceed. Only when the solvation sheath starts to lose solvating members could the reduction of Mn^2+^ become possible (Fig. 3c), where the electron affinity energy of Mn^2+^ eventually approaches that of EC with decreasing solvation number. Hence the opportunity of Mn^2+^ reduction cannot be completely ruled out when anode potential is driven under certain threshold values, where strong electric field would strip Mn^2+^ off any solvent molecules or anions, and the low potential will force Mn^2+^ to reduce. This explains why metallic Mn was rarely detected on graphite surface, but occasionally evidences were still presented for their existence^31–33^. Alternative two-electron pathway was also considered, where the reduction activity and reduction structures of Mn^2+^ solvation sheath, in presence and absence of PF_6_^−^, were shown in Supplementary Figs. 10 and 11. It can be further noted that the electron affinity energy of Mn^2+^ reduction is always lower (less negative) in comparison with the reductions of EC and PF_6_^−^ in a solvation sheath, indicating that the possibility of Mn^2+^ to receive two electrons is even less likely than the scenario of one-electron reduction of Mn^2+^ (Fig. 3c, d). Similarly, reduction of PF_6_^−^ anion remains the most thermodynamically favorable reaction.

To further examine the previous belief that TM particles in elemental form (Mn^0^) catalyze the reduction of carbonate molecules on electrode surface, the ability of Mn^0^ and Mn^2+^ in activating EC for reduction is compared (Fig. 3f), with the corresponding optimized structures shown in Supplementary Fig. 12. The electron affinity of EC-Mn^2+^ is obviously higher (more negative) than other investigated complexes, Mn^0^ in particular, which implies that Mn^2+^ is more effective in inducing electrons from the anode and then mediate it to carbonate molecules for reduction. This should be apparently attributed to the stronger electron-withdrawing capability of Mn^2+^ than Mn^0^. The electron affinity of EC-Mn^0^, though, is still higher than the neat EC solvent, therefore the existence of metallic Mn indeed would activate EC for reduction, but should not constitute the major contribution when Mn^2+^ is present.

**Fig. 4:**
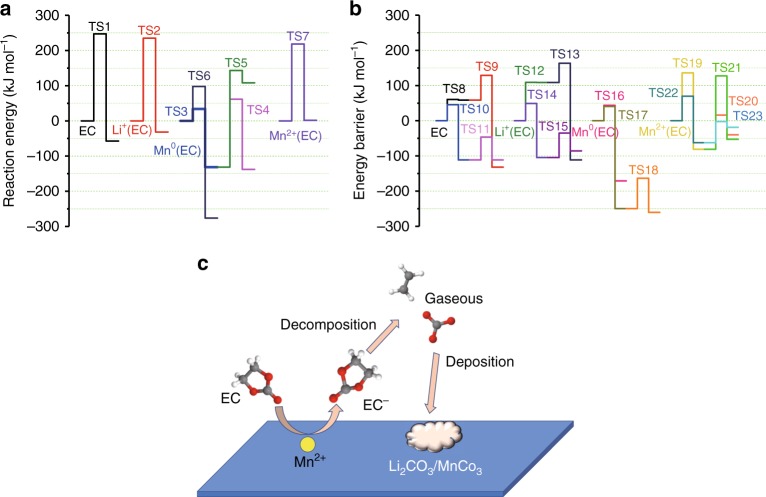
Influence of Mn^2+^ species on the decomposition mechanism of electrolyte. Decomposition reaction profiles of EC with and without interacting with Li^+^, metallic Mn and Mn^2+^ before (**a**) and after one electron reduction (**b**) from DFT calculation; Catalytic mechanism of Mn^2+^ on the reduction decomposition of electrolytes (**c**)

The similar trend can be derived from the energy profiles of EC decomposition in the absences or presences of Li^+^, Mn^0^, and Mn^2+^ (Fig. 4), respectively, with the optimized structures shown in Supplementary Figs. 13 and 14. The existence of Mn^0^ significantly lowers the EC decomposition activation energy (Fig. 4a) from 247.20 (TS1) to 33.69 (TS3) kJ mol^−1^. Interestingly, the decomposition activation energy of EC coordinated with Mn^2+^ (TS7) is lower than that of neat EC (TS1) by only 16.57 kJ mol^−1^. On the other hand, once receiving one electron, the reduced form of EC decomposition (TS10) becomes significantly easier than EC (TS1) before reduction. In consideration of the strong catalytic effect of Mn^2+^ on EC reduction (Fig. 3f), the reduced form of EC may dissociate from Mn^2+^ and then proceed with further cascade of decomposition (TS10) without Mn^2+^. The decomposition activation energy of reduced EC with Mn^2+^ (TS21) is about 9 kJ mol^−1^ higher than that of reduced EC (TS10), again indicating that after being receiving one electron, EC anion would dissociate from Mn^2+^. The leaving EC molecule would leave a coordination vacancy near Mn^2+^ for other incoming EC molecules. In an overall picture of the entire catalytic process (Fig. 4c), the Mn^2+^-solvation sheath, no matter in bulk electrolyte or on electrode surface, acts as the catalytic center, which continuously activates the carbonate molecules for reduction, leading to incessant electrolyte consumption and cell degradation. On atomic level, one would expect that such activation of solvation sheath members by the center TM cation should be directly related to the specific d-electron configuration of the latter. This realization carries significant importance to the understanding of the electrolyte destabilization mechanisms, and should be further explored.

Finally, it should be cautioned that the Mn-content deliberately added in this work is higher than an electrolyte could experience in actual battery, hence the effect of Mn-catalysis may have been exaggerated. However, for system of extreme complexity such as the electrolyte decomposition, exaggeration more often than not brings necessary simplicity that serves as starting point to derive a model that can be further perfected. The mechanism and fundamental understanding achieved in this work therefore help shed initial light on an issue that was under-investigated because of its difficulty.

## Discussion

We demonstrated in this work that, besides interfacial Mn-species, the once overlooked Mn^2+^ in bulk electrolyte solution significantly contributes to destabilize the carbonate-based electrolytes. In comparison with Li^+^, the bivalent Mn^2+^ generates larger solvation sheath containing both PF_6_^−^ and carbonate molecules, with cyclic EC preferred over acyclic DMC. Within such intimate distance confined by the solvation sheath, Mn^2+^ readily destabilizes both carbonate molecules and anion, either for side reactions in the bulk or for electrochemical reduction at anode surface. The destabilization of bulk electrolyte become rather pronounced at elevated temperatures, which could have significantly changed the bulk electrolyte composition. In particular, at the anode surface, Mn^2+^-solvation sheath activates its solvating members (carbonate molecule, anion) for reduction, rather than being reduced into Mn^0^. The presence of Mn^2+^ in bulk electrolyte or on anode surface serves as catalytic centers that induce continuous electrolyte consumption at the expense of the cell performances.

## Methods

### Molecular dynamic simulations

The electrolyte was modeled with the nonpolarizable force-field from refs. ^34,35^ for Li^+^, PF_6_^−^, EC, DMC, and TFSI^−^, and Bradbrook et al.^36^ for Mn^2+^. The starting configurations were prepared utilizing fftool^37^ and packmol^38^. The initial setup electrolytes were equilibrated at 393 K and 1 atm for 2 ns in NPT ensemble. The production runs were generated in NVT ensemble at 393 K and had a length of 168.4 ns for the system with Mn^2+^ and 20 ns for simulations without Mn^2+^. Nose Hoover thermostat was adopted to control the temperature^39^ having a coupling constant of 200 fs. The van der Waals and real space Ewald short-range interactions were calculated within a 12 Å spherical cut off. The electrostatic interactions were calculated utilizing Ewald method^40^ and particle mesh scheme^41^ for the reciprocal part. The coordination numbers around ions were calculated at the following distances (encompassing most of the first peak in rdf) ion-O = 2.4 Å, ion-P = 4.4 Å, ion-F = 2.8 Å.

The simulation cell was cubic and had 3D periodicity. The simulated systems consisted of 306 molecules of DMC and 312 molecules of EC (1:1 weight ratio), 48 ionic pairs of LiPF_6_ and 6 ionic pairs of Mn(TFSI)_2_.

### Density functional theory calculations

All DFT calculations were performed with the Gaussian 09 software^42^. B3LYP/6-311 + + G (d) method was adopted to optimize the investigated solvents and complexes^26,43^. Frequency analysis was applied with the same method to verify that each obtained optimized structure was at the energy minimum point. The energy shown in this work were calculated at 298.15 K. Solvent effects were considered with the application of polarized continuum models using a dielectric constant of 20.5 (Acetone solvent). Charge distributions were analyzed by nature population analysis according to natural bond orbital (NBO) theory. 3*d*^5^ was chosen as the outmost electrons in DFT calculation for Mn^2+^.

### Sample preparation

Battery-grade ethylene carbonate (EC), dimethyl carbonate (DMC) and lithium hexafluorophosphate (LiPF_6_) were provided by Guangzhou Tinci Materials Technology Co. Ltd., China. Lithium bis(trifluoromethanesulfonyl)imide (LiTFSI) (99%) and manganese (II) bis(trifluoromethanesulfonyl)imide (Mn(TFSI)_2_) (>98%) were provided by Aladdin. The above electrolyte components were used without further refinement. A base solvent, EC/DMC (1/1 in weight) was formulated. 1.0 M LiPF_6_, 0.6 M LiTFSI, 0.3 M Mn(TFSI)_2_, [1.0 M LiPF_6_ + 0.6 M LiTFSI] and [1.0 M LiPF_6_ + 0.3 M Mn(TFSI)_2_] were applied in the base solvent to obtain electrolyte of 2, 3, 4, 5, and 6 respectively, see Supplementary Table 1. The preparation of the electrolyte is carried out in the glove box (MBraun, Germany) filled with argon, in which the O_2_ and H_2_O contents were lower than 10 and 0.1 ppm, respectively. The H_2_O content of electrolytes were measured by Karl-Fisher 798 GPT Titrino (Metrohm, Switzerland). The H_2_O content shown in this paper were the average of three parallel tests.

The injection of 1000 ppm HF, whose effect was shown in Supplementary Fig. 3, was prepared in two steps. At first 2.1 mL 20 M L^−1^ HF solution was injected into 40 mL Electrolyte 1 to obtain HF-containing baseline. Then 3 μL HF-containing baseline was respectively injected into 3 mL Electrolytes 1, 2, 3, 4, 5, and 6 to obtain the samples with 1000 ppm HF.

Graphite slurry was obtained by mixing artificial graphite (80 wt%, offered by Dongguan Kaijin New Energy Technology Co., Ltd, China), acetylene carbon black (10 wt%) and poly(vinylidene fluoride; 10 wt%) in *N*-methyl-pyrrolidone. The above slurry was then coated on Cu current collector and dried at 80 and 120 °C for 2 and 12 h in vacuum drying oven, respectively, to obtain graphite electrode. The graphite electrode was rolled before used. Lithium plate was used as counter and reference electrode in CR2025-type graphite/Li half-cells with Celgard 2400 membrane separator. Cell assembly was completed in the glove box.

### Electrolyte storage

The prepared electrolytes were initially stored in polyethylene bottles sealed with an air-tight lid, which was further sealed with teflon tape and cellophane tape to ensure the good sealing (see Supplementary Fig. 15). This sealing has been adopted by some electrolyte companies when shipping solvents and electrolytes. The polyethylene bottles were transferred subsequently to the vacuum dryer for storage. After 8 days of storage at high temperature, the plastic bottles were transferred to the glove box again. And the stored electrolytes were finally transferred into glass bottles for photograph because the polyethylene bottles are opaque.

To further verify the airtightness/moisture-exclusiveness of the plastic bottles and sealing, we measured the H_2_O content of electrolyte 1 (solvents only, with no influence from salts) before and after 8-day storage in the vacuum dryer, which turned out to be 20 and 21 ppm, respectively, confirming that the manner that electrolytes are sealed and stored could effectively exclude the permeation of moisture from ambient.

### Electrochemical measurements

The room-temperature cyclic stability of Li/graphite half-cells were tested between 0.005 and 2.5V with LAND system (CT2001A, Wuhan, China). After 3 cycles of activation at 0.1C, the Li/graphite half-cells were performed at 0.2C (1C = 372.6 mA g^−1^) for the subsequent cycles.

### Physical characterization

The electrospray ionization mass spectrometries (ESI-MS) of electrolyte before and after storage at 55 °C were executed on TSQ Quantum Ultra. The spray voltage was +3 kv while the capillary temperature was 300 °C. The sheath gas and aux gas flow rates were 30 and 5 arb, respectively. Methanol was used as a co-solvent during the ESI-MS characterization. The date ranging from m/e = 50–1000, were available. Thermal stability of electrolytes in the temperature range of 25–300 °C was investigated using differential scanning calorimeter (DSC, PerkinElmer DSC 4000, Hetherlands) with 10 °C min^−1^ heating rate.

Ex-situ scanning electron microscope (SEM) and transmission electron microscope (TEM) characterization were performed on JEOL-5900 SEM and JEM-2100HR (Japan), respectively. All electrode samples were vacuum transferred to the test equipment. The acidity of electrolytes after storage were measured by precise PH test papers (PH:1.4–3.0, Q/GHSC) which were provided by Shanghai SSS Reagent CO. LTD, Shanghai China. Gaseous products of electrolyte decomposition during storage were investigated by Gas chromatography mass spectrometry (GC-MS) QP2010 Ultra Shimadzu. Approximately 10 mL Electrolytes 5 and 6 were stored in pouch bags made from the same foil materials used to assemble pouch cells (see Supplementary Fig. 16).

After 8-day storage at 55 °C, ~0.8 mL gas sample was extracted from the bags with a syringe through the pre-installed valves and then injected into a DB-5 capillary column (30 m × 0.25 mm inner diameter, 0.25 μm film thickness). Carrier gas, Helium, was used at a flow rate of 1.47 mL min^−1^. The inlet temperature was 150 °C, while the column temperature was initially set at 30 °C for 10 mins, then increased to 150 °C with 10 °C min^−1^ and maintained for another 5 mins. The mass spectrometer was operated with electron impact (EI) mode (0.1 kV) at the rate of 1250 amu s^−1^. Full-scan mass spectra were obtained from 20 to 100 m/e. The MS results were shown in Supplementary Fig. 4.

## Supplementary information

Supplementary Information

## Data Availability

The authors declare that the main data supporting the findings of this study are available within the article and its Supplementary Information files. Extra data are available from the corresponding author upon request.

## Code availability

The molecular dynamic simulations were run utilizing the LAMMPS^44^ general purpose open-source code, and according to LAMMPS website https://lammps.sandia.gov, “LAMMPS is distributed as an open source code under the terms of the GPL”. The code LAMMPS can be downloaded from: https://lammps.sandia.gov/download.html.
