# Supplementary material for: Overlooked electrolyte destabilization by manganese (II) in lithium-ion batteries
Source: Nat Commun. 2019 Jul 31;10:3423. doi: 10.1038/s41467-019-11439-8 (PMC6668472; doi:10.1038/s41467-019-11439-8)
Supplement: Supplementary file 1 — Supplementary Information [file 41467_2019_11439_MOESM1_ESM.pdf]

Supporting information for:

Overlooked Electrolyte Destabilization by Manganese(II) in Lithium-ion Batteries

Wang et al.

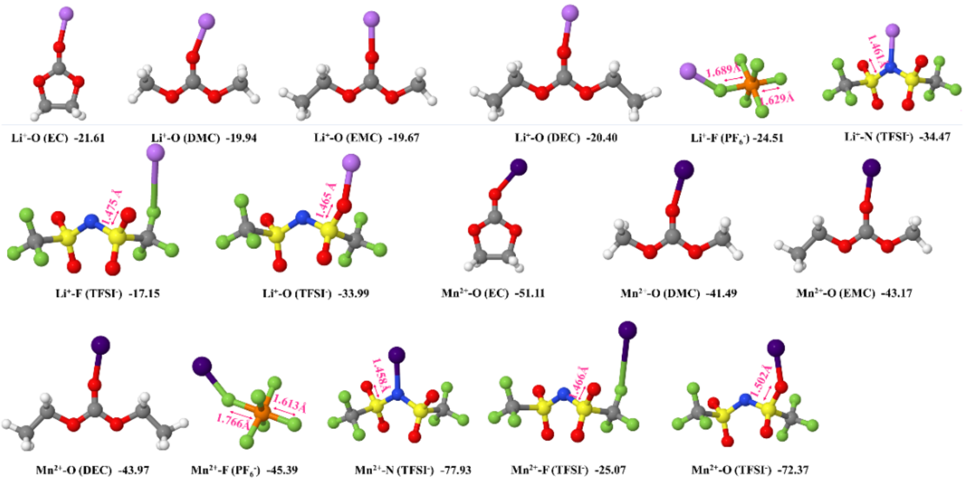

Supplementary Figure 1. Optimized structures and interaction energy of Li^+/Mn^{2+} with solvent and salt anion. Optimized structures of Li^+ and Mn^{2+} interacts with ethylene carbonate (EC), dimethyl carbonate (DMC), ethyl methyl carbonate (EMC), diethyl carbonate (DEC) solvent and PF_6^-, TFSI$^-$ anion obtained from DFT calculation, together with the corresponding interaction energy (in kJ mol^{-1}).

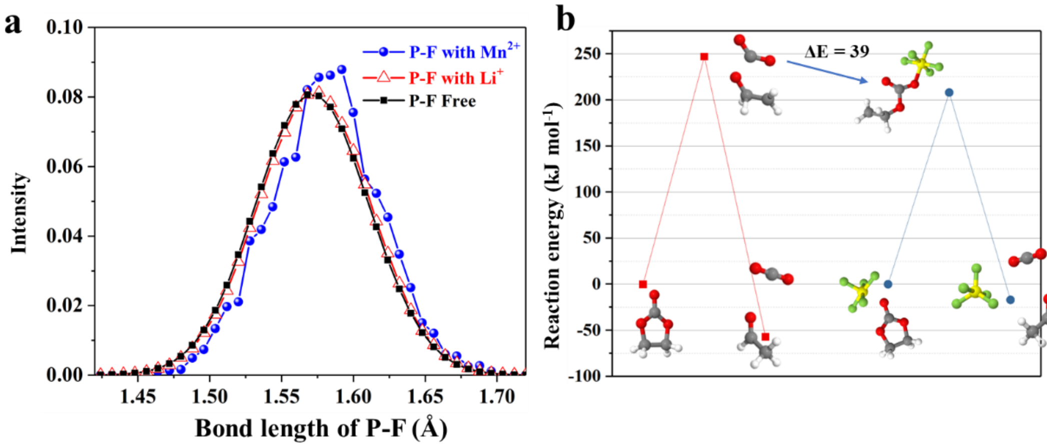

Supplementary Figure 2. Structure change of PF_6^- and influence of PF_5 on the decomposition mechanism of EC. P-F bond length distribution in PF_6^- with and without interacted with Mn^{2+} and Li^+ (a); influence of PF_5 on the decomposition of EC solvent from DFT calculation (b).

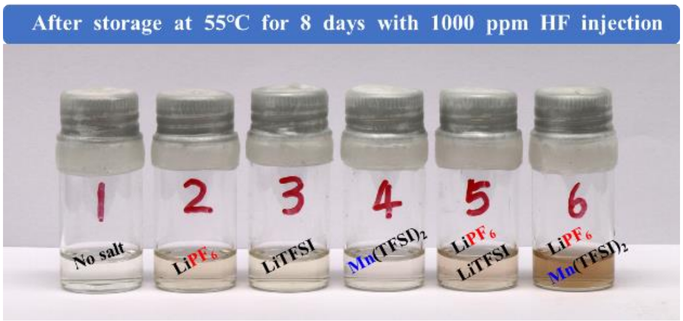

Supplementary Figure 3. 1000 ppm HF injection-electrolytes after storage at 55 °C for 8 days.

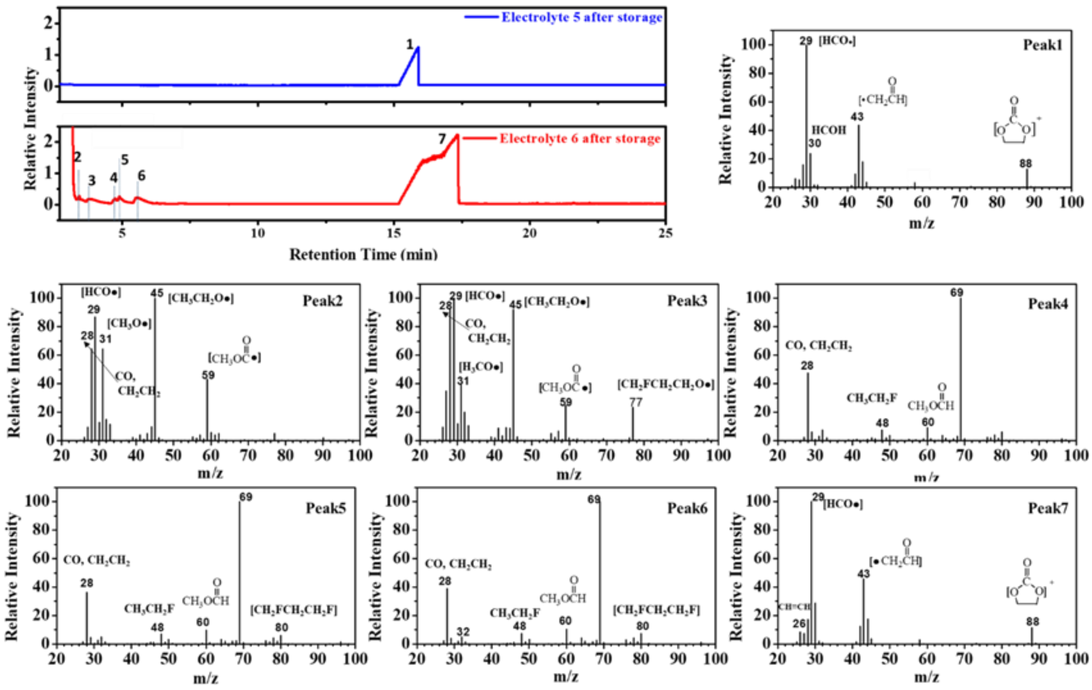

Supplementary Figure 4. GC/MS chromatograms of 0.8 mL gas products of Electrolyte 5 and 6 after storing at 55℃ for 8 days.

Supplementary Note. The picture shown in Supplementary Figure 5 were obtained by dropping 15 μL electrolytes after storage on a dry high precision PH test paper (1.4-3.0).

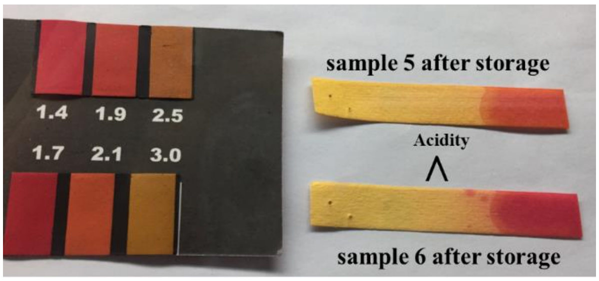

Supplementary Figure 5. Acidity of Electrolytes 5 and 6 after storage characterized by high precision PH test paper (1.4-3.0).

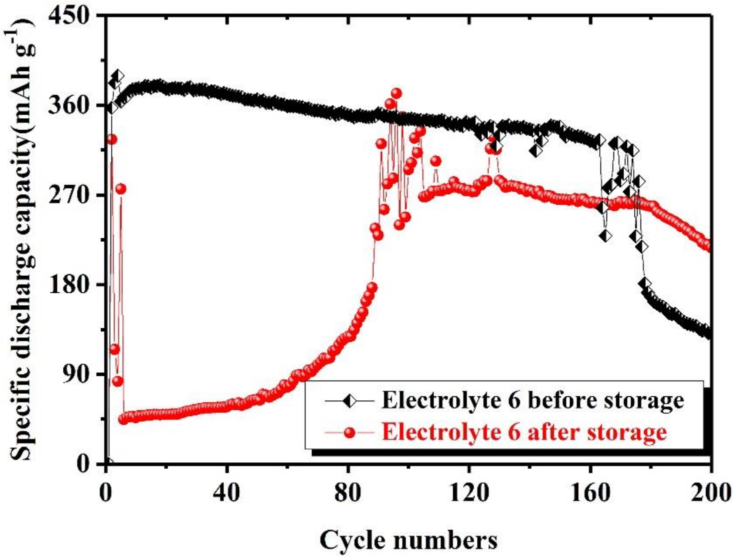

Supplementary Figure 6. Cyclic stability of graphite/Li half-cell between 0.005 to 2.5 V vs. Li/Li^+ with Electrolytes 6 before and after storage.

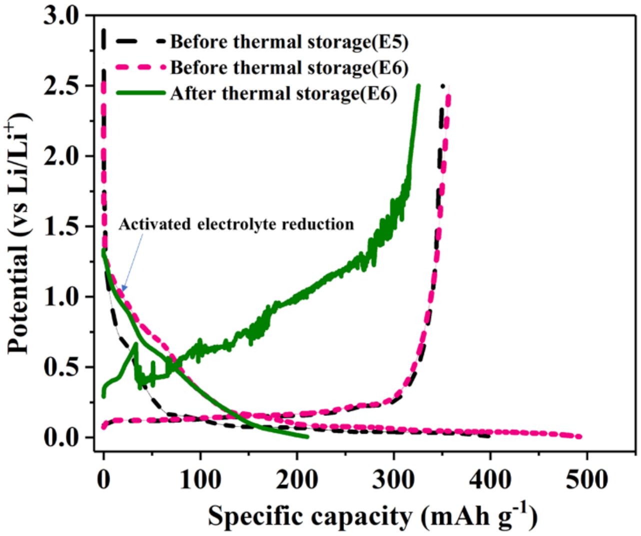

Supplementary Figure 7. Initial charge/discharge curves of graphite/Li half-cell. Initial charge/discharge curves of graphite/Li half-cell with fresh Electrolyte 5, and Electrolyte 6 before and after storage at 55°C for 8 days; the cells were charge/discharge at 0.1C between 0.005 to 2.5 V vs. Li/Li^+.

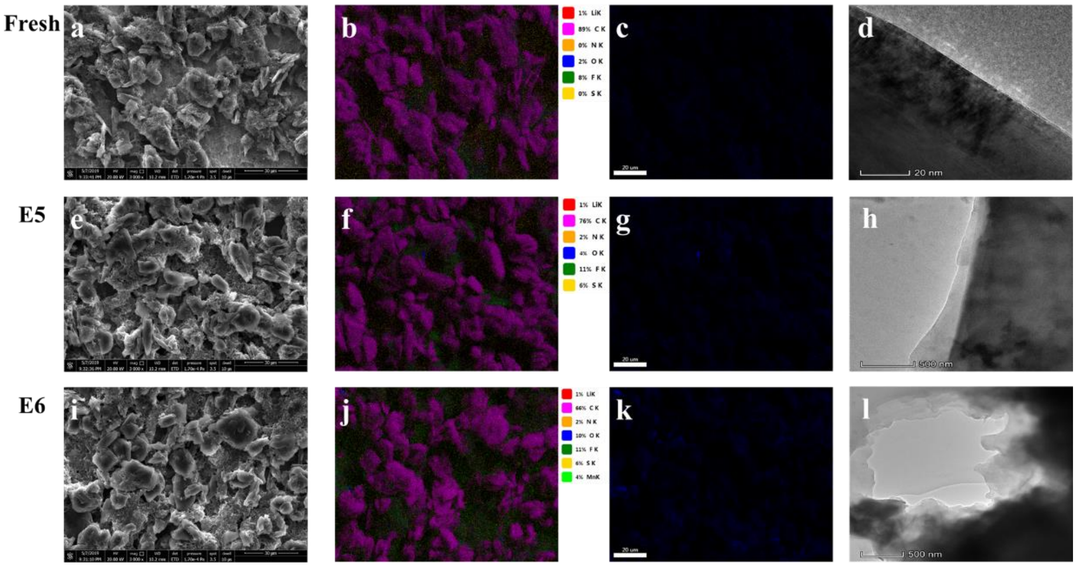

Supplementary Figure 8. Surface morphology and composition of graphite electrodes. SEM images and the corresponding elemental mapping, and TEM images of fresh graphite (a, b, c and d) and graphite after cycling with fresh Electrolyte 5 (e, f, g and h) and fresh Electrolyte 6 (i, j, k and l) between 0.005 to 2.5 V vs. Li/Li^{+}.

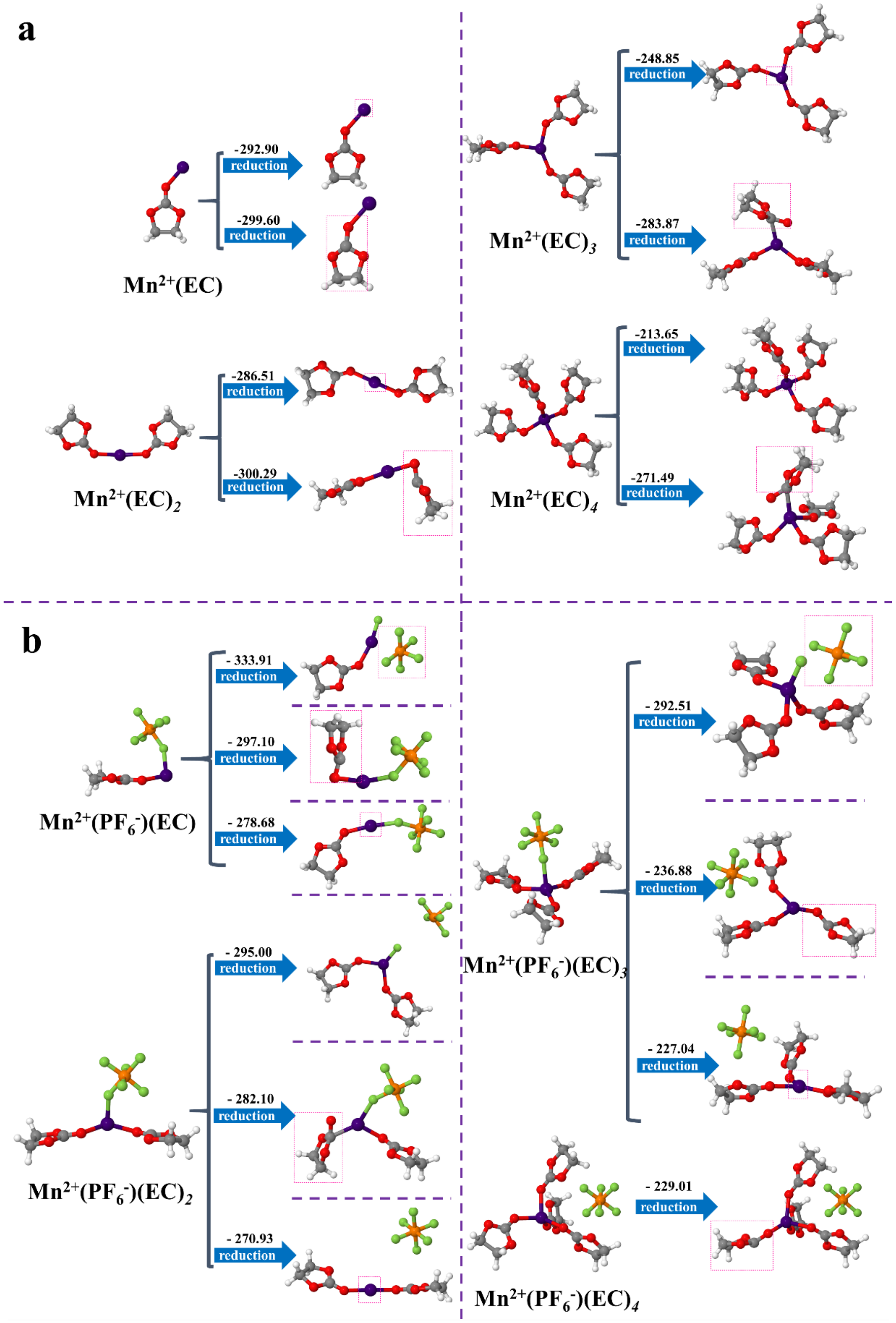

Supplementary Figure 9. One electron reduction of Mn^{2+} solvation shells. Optimized structures of Mn^{2+} solvation shells without (a) and with PF_6^- (b) after one electron reduction; together with the corresponding electron affinity energy (in kJ mol^{-1}); Species that gaining electron are highlighted with graticule line.

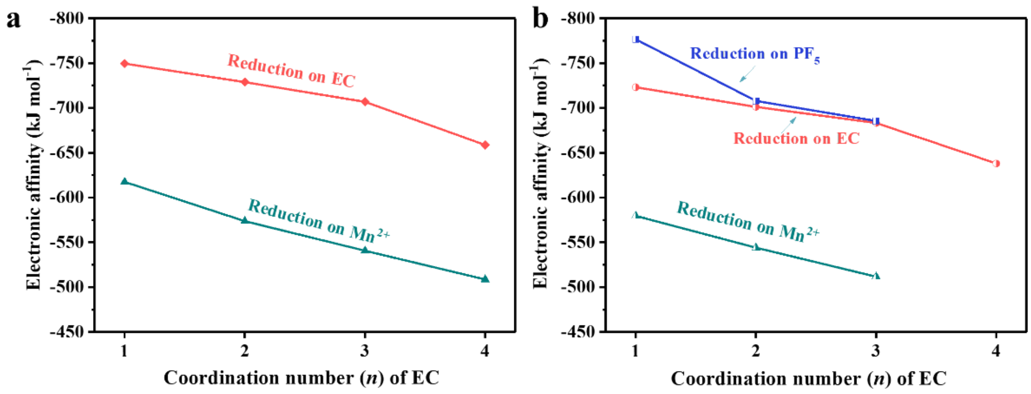

Supplementary Figure 10. Electron affinity energy of Mn^{2+} solvation-shells. Calculated electron affinity energy of $Mn^{2+}(EC)_n$ solvation-shells without (a) and with (b) PF_6^- in two-electron reaction.

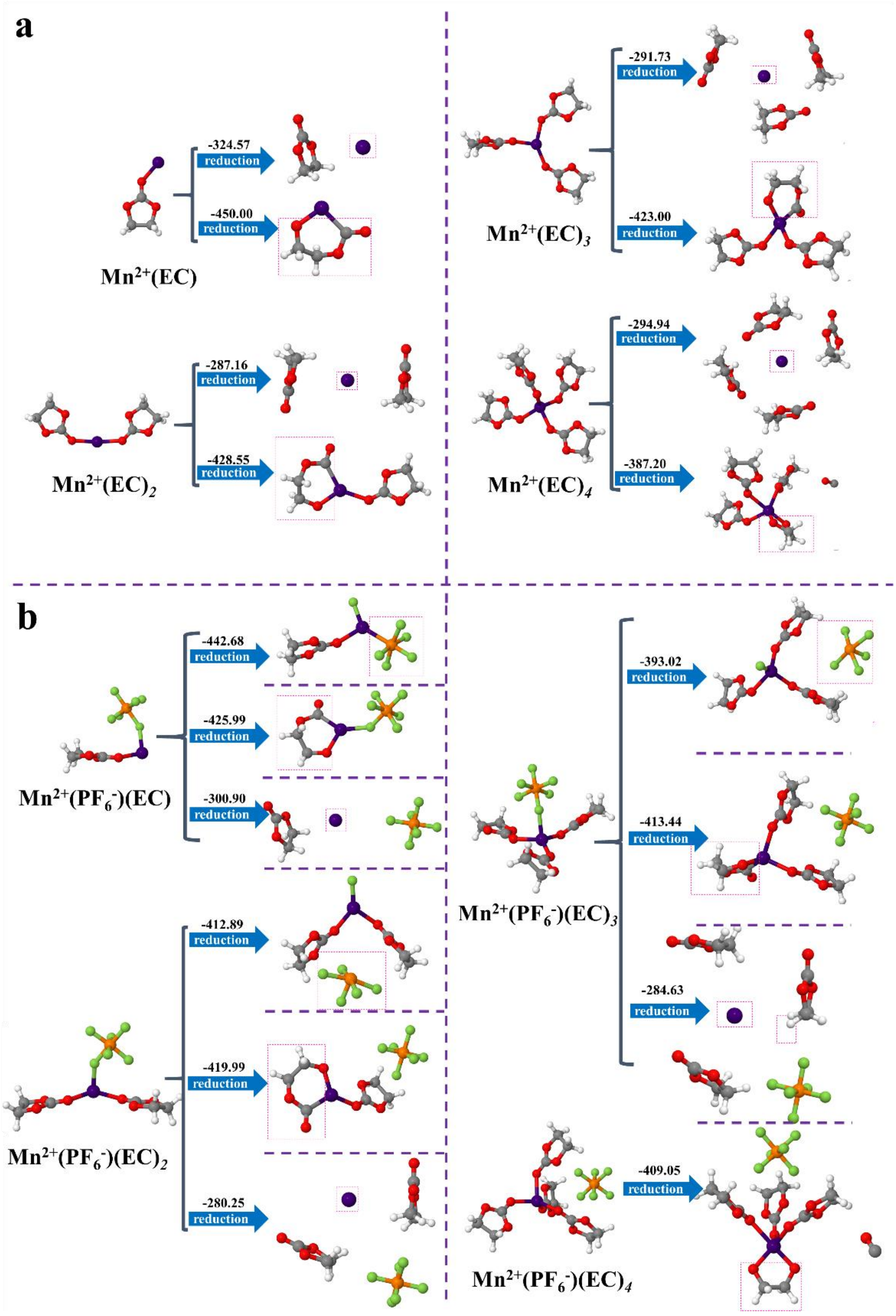

Supplementary Figure 11. Two electrons reduction of Mn^{2+} solvation shells. Optimized structures of Mn^{2+} solvation shells without (a) and with PF_6^- (b) after two-electron reduction, together with the corresponding electron affinity energy (in kJ mol^{-1}). Species that gaining electron are highlighted with graticule line.

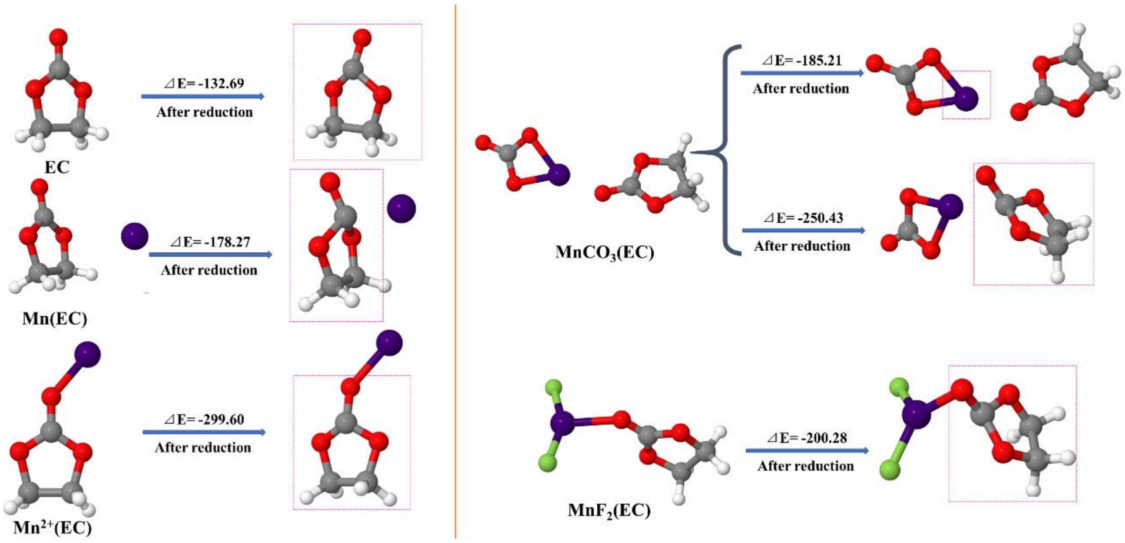

Supplementary Figure 12. Influence of Mn species on the reduction reaction of EC solvent. Optimized structures of EC with Mn, Mn^{2+}, $MnCO_3$ and MnF_2 before and after one electron reduction, together with the corresponding electron affinity energy (in kJ mol^{-1}). Species that gaining electron are highlighted with graticule line.

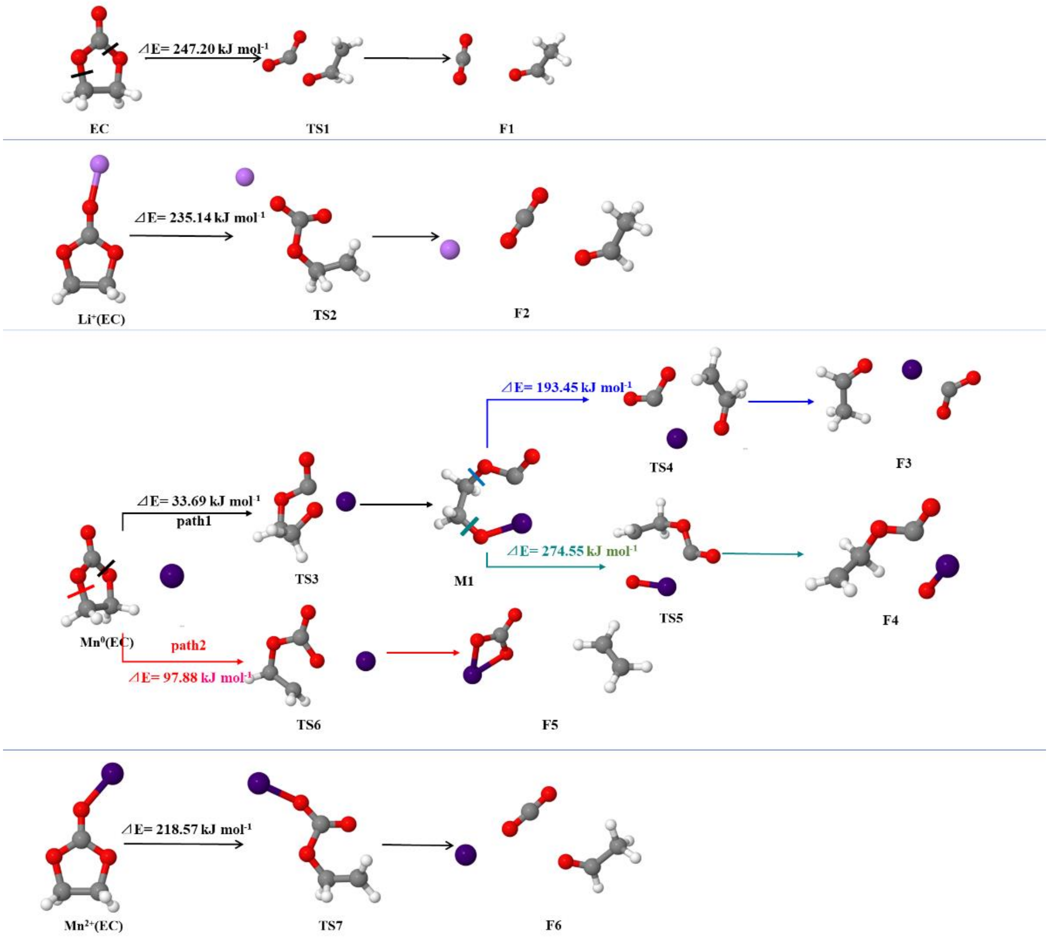

Supplementary Figure 13. Decomposition mechanism of EC solvent with various species. Optimized structure of EC decomposition without and with Li^+, Mn^0 and Mn^{2+}, together with the reaction energy (in kJ mol^{-1}).

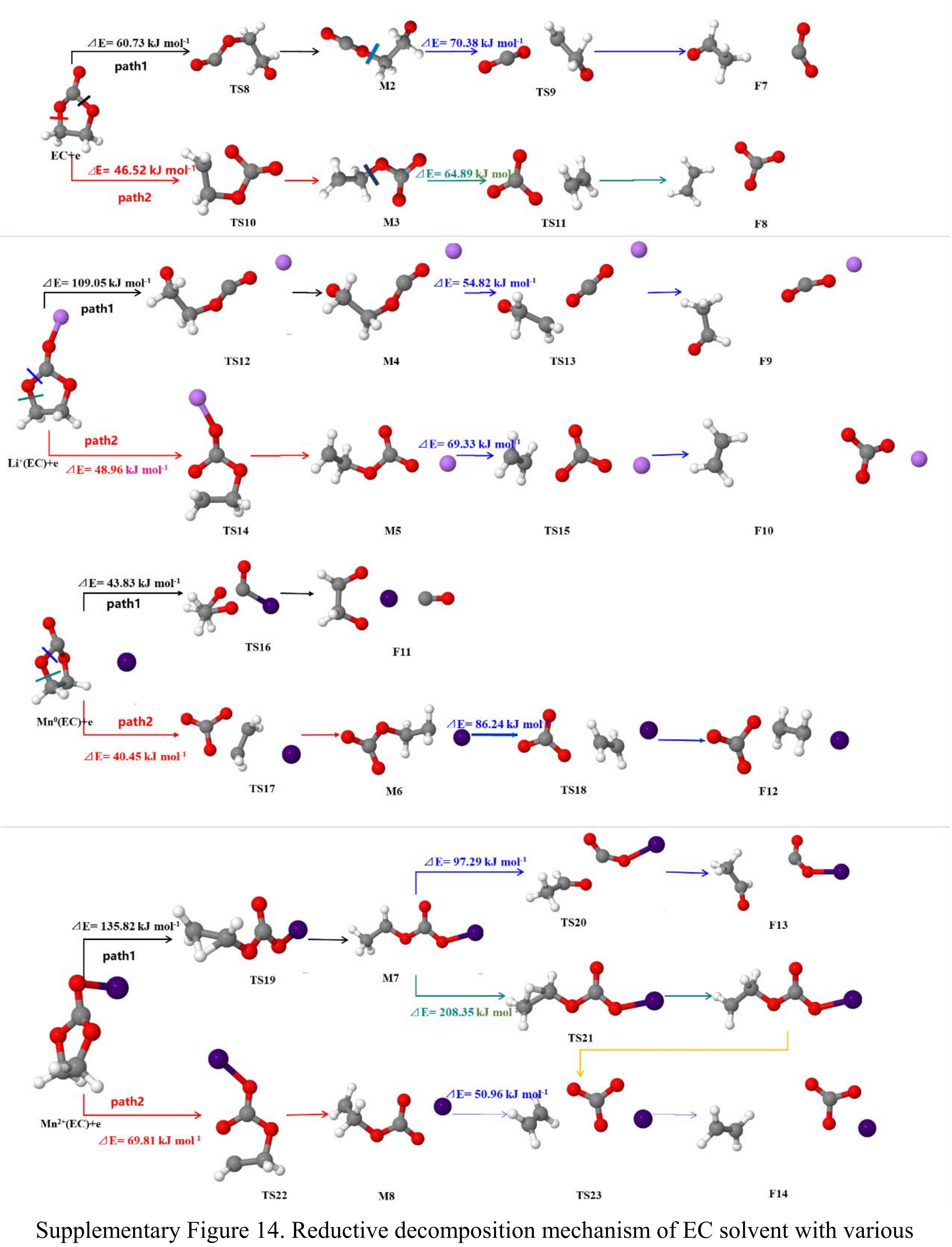

Supplementary Figure 14. Reductive decomposition mechanism of EC solvent with various species. Optimized structure of EC decomposition without and with Li^{+}, Mn^{0} and Mn^{2+} after one electron reduction, together with the reaction energy (in kJ mol^{-1}).

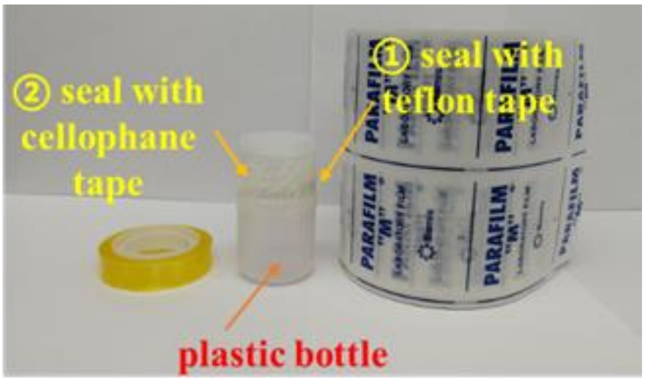

Supplementary Figure 15. Schematic diagram of electrolyte sealing.

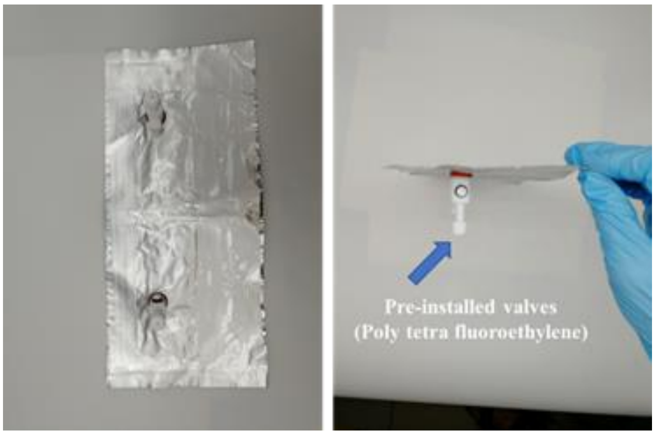

Supplementary Figure 16. Electrolytes stored in pouch bags for GC-MS characterization.

Supplementary Table 1. Electrolyte formulations.

Electrolytes	formulation
1	EC/DMC (1:1 by weight)
2	1.0 M $LiPF_6$ in EC/DMC (1:1 by weight)
3	0.6 M LiTFSI in EC/DMC (1:1 by weight)
4	0.3 M MnTFSI in EC/DMC (1:1 by weight)
5	1.0 M $LiPF_6$ +0.6 M LiTFSI in EC/DMC (1:1 by weight)
6	1.0 M $LiPF_6$ +0.3 M MnTFSI in EC/DMC (1:1 by weight)
